# Evaluation of Biopolyurethane/Barley Straw-Based Engineered Wood Composites

**DOI:** 10.3390/polym18111312

**Published:** 2026-05-26

**Authors:** Sigitas Vėjelis, Ugnė Kornelija Aglinskaitė, Arūnas Kremensas, Saulius Vaitkus, Jurga Šeputytė-Jucikė, Aurelija Rimkienė

**Affiliations:** Building Materials Institute, Faculty of Civil Engineering, Vilnius Gediminas Technical University, Linkmenų Str. 28, LT-08217 Vilnius, Lithuania

**Keywords:** polyurethane adhesive, agricultural waste, pressing of mixture, engineered wood, physical–mechanical properties, microstructure

## Abstract

More than 95% of building materials in Europe are produced from fossil raw materials. Over the past two decades, numerous scientific studies have demonstrated that building materials made from agricultural plants or industrial processing waste can compete with traditional materials. In this work, engineered wood composites were prepared from biopolyurethane and barley straw, and their properties were evaluated. Barley straw from bales was milled through sieves of different sizes. Four straw fractions of 5, 10, 25 and 35 mm were prepared for testing. During the research, the granulometric composition, particle density and shape of various fractions were evaluated. Engineered wood composites were prepared using different filler fractions and a biopolyurethane binder. In this study, engineered wood samples were produced using biopolyurethane binders at straw-to-binder ratios of 0.5 to 1.5. Different pressure levels were used for sample preparation: 1.5, 2.25 and 3.0 MPa. This study evaluated the influence of the granulometric composition and particle shape of straw on the properties of engineered wood composites. Tests showed that the highest compressive strength, 17.0 MPa, was achieved with composites formed from a 5 mm straw fraction, which had the highest density. The samples with the highest density were also characterised by the lowest swelling (5–10%) and water absorption (1–2%). The flammability of the samples showed that at a 0.5 binder/straw ratio, the composite was non-combustible and did not support flame spread after the flame source was removed.

## 1. Introduction

Every year, large amounts of organic waste are generated in various industrial sectors. The range of this waste is very wide: animal and mixed food waste, wood waste, plant waste, paper and cardboard waste and mixed waste [1]. In Lithuania, the largest amount of all this waste is straw [1]. Straw, as an agricultural by-product, causes significant environmental problems due to its high production volume and low utilisation efficiency. Identifying low-carbon, scalable utilisation methods remains a major challenge worldwide [2]. The resulting straw can be used or disposed of in various ways. Straw can be harvested from the field and used for feed [3,4], bedding [5,6,7], compost [8,9], gas production [2], milled and mixed with soil [10,11,12], burned in the field [13,14] or collected and used for biofuel [15,16]. There is a wealth of evidence for the use of straw in the production of building materials [17,18,19,20]. The use of straw as a building material dates back to ancient times and has experienced a revival in recent years, reflecting the increasing focus on sustainable building practices. As a renewable resource, readily available straw offers a cost-effective alternative to traditional building materials such as concrete, steel and brick [21]. Straw, as an agricultural by-product, can be used as a thermal insulation material in the form of whole-straw bales, blown insulation, or composites [22]. Straw is most used in building construction as rectangular bales and has been evaluated for its thermal conductivity and durability [23]. The first straw bale houses were typically constructed from wheat, rice or barley straw, depending on local availability [24]. Straw bale buildings have several disadvantages: their use involves unconventional construction processes; uncertainty regarding the repeatability of physical parameters; risk of biological corrosion, fire, and pests; low building tightness; and risk of water vapour condensation in external walls [25]. Straw bale buildings are usually constructed using a wooden frame in which the bales are firmly anchored [26]. Another way to use straw is to spray chopped straw onto vertical or horizontal surfaces [27]. The use of chopped straw in building structures has a great advantage, as it can be treated with various additives that reduce flammability [28] and increase mould resistance [29]. The use of straw to produce composites involves several technological processes, including binder selection and production technologies. Straw is treated with various means to remove soluble components covering its surface, such as waxes, lignin and hemicellulose, and to improve the interface with the binder [30,31,32]. The most common pretreatment methods for natural fibres are steam cooking, steam explosion, physical radiation, liquid hot water and chemical treatment [33]. Of the chemical treatments, alkali treatment is the most used. When treating with alkali, excessively high alkali concentration or temperature can lead to significant deterioration in fibre properties due to excessive delignification [34]. By selecting the appropriate chemical treatment regime, it is possible to reduce the water absorption rate, improve the processability of straw materials, and improve the physical and mechanical properties of composite materials [35]. Depending on the chemical composition of the straw, a binderless board production process can be used, or an appropriate binder can be selected. The chemical composition of the raw materials varies depending on the type of material and different parts of the plant [36]. Binderless boards, produced without glue, exploit the natural bonds among lignocellulosic components during hot pressing [37]. These bonds include lignin softening, thermoplasticisation and covalent cross-linking. Although the produced boards are environmentally friendly, they also have disadvantages. Their production involves heat treatment, which takes place at a temperature of 150–180 °C [38,39]. In addition, with heat treatment, board thickness is limited because, during curing at high temperatures, the outer layers bond quickly, while the middle layers take significantly longer to bond. Thus, when producing thicker boards, the outer layer starts to break down due to pyrolysis, while the middle layer does not fully bond, resulting in an inhomogeneous product [40].

The synthetic adhesives most used in the production of fibreboards are formaldehyde-based [40] and derived from non-renewable resources. They are classified as carcinogenic to humans by the International Agency for Research on Cancer and classified as Category 1B [41]. For all the above, the use of non-toxic and bio-based binders such as starch, soybean, lignin, chitosan, and, more recently, cellulose nanofibers has been widely studied as an alternative to formaldehyde-based binders [42]. Both formaldehyde-based resins and bio-based binders require heat treatment and limit product thickness. Several scientific studies used biopolyurethane as a binder [43,44,45,46]. The curing of the biopolyurethane binder occurs due to the reaction of hydroxyl groups with isocyanate groups [47]. In this way, without heat treatment, it is possible to produce products of great thickness while ensuring their homogeneity. In addition, a large amount of fossil raw materials is replaced by natural biopolyols obtained from plant pulp [48].

The aim of this work is to evaluate the possibilities of creating an engineered wood composite with high-performance properties using biopolyurethane and agricultural waste—barley straw—without using thermal treatment.

## 2. Materials and Methods

### 2.1. Raw Materials

The composite filler was selected from barley straw grown in eastern Lithuania. For research and composite formation, the straw was delivered in bundles measuring 700 × 500 × 400 mm. To prepare particles of the required size, the straw was milled with a straw mill using sieves with mesh sizes of 5, 10, 25 and 35 mm. The obtained straw fractions were used to study their properties and to produce composites. A knife-type hay and straw mill, SNR 30 (TechnoMashStroi, Cherkasy, Ukraine), with replaceable sieves of 5, 10, 25, and 35 mm, was used for straw milling. The mill engine power was 1.1 kW, the rotational speed was 3000 rpm, the number of knives was 4 pcs., and the capacity was 50 kg/h.

To increase adhesion between the filler particles and the binder and to evaluate the effects of each treatment, the milled straw was treated with water, sodium carbonate (Na_2_CO_3_), and sodium silicate (Na_2_SiO_3_).

In water treatment, the milled straw was soaked in boiling water until fully covered and left for 24 h. After 24 h of immersion, the straw was drained and washed six times with water at a temperature of (23 ± 5 °C). The washed straw was placed on shelves in a 30 mm thick layer and dried in a drying oven to a constant weight at 70 °C.

In the sodium carbonate treatment, the milled straw was poured with sodium carbonate (1000 g of milled straw–200 g of sodium carbonate) and boiling water. The straw prepared in this way was left to soak for 24 h. After the immersion time, the straw was drained and washed six times with water at a temperature of (23 ± 5 °C). The washed straw was placed on shelves in a 30 mm thick layer and dried in a drying oven to a constant weight at 70 °C.

When treated with sodium silicate, the milled straw was immersed in boiling liquid glass solutions at 10% and 30% concentrations and soaked for 10 min. After immersion, the straw was drained, placed on shelves in a 30 mm thick layer, and dried in a drying oven at 70 °C until a constant weight was reached.

The composite binder was chosen as a bio-based polyurethane consisting of rapeseed oil, a biopolyol, and an isocyanate. Polylabs (Riga, Latvia) BioPolyol RD polyol from rapeseed oil was used for the preparation of the binder, which is mainly used in the production of rigid polyurethane foams (density 976 kg/m^3^, viscosity at 25 °C—800–1000 mPa s, hydroxyl group (OH) content—360–410 mgKOH/g). As a hardener for binder preparation, Lupranat M20 S 4,4′-dimethylmethane diisocyanate, manufactured by BASF, Ludwigshafen, Germany (viscosity at 25 °C—220 mPa s; density at 25 °C—1.22 g/cm^3^), was used to produce insulating and rigid polyurethane foams. Refined, deodorised rapeseed oil (UAB Lomista, Kaišiadorys, Lithuania) was used to increase the binder volume and reduce its viscosity. The density of rapeseed oil was 0.912 g/cm^3^ and 65.5 at 25 °C, and the content of hydroxyl groups (OH) was 182–193 mgKOH/g.

To improve the resistance of the composite from milled straw to fire, water and atmospheric influences, property-improving additives were used. Expandable graphite was used to increase fire resistance (manufacturer Qingdao Kropfmuehl Graphite Co., Ltd., Qingdao, China); particle size—more than 80% larger than 300 µm; expansion volume ≥ 350 cm^3^/g). This additive, used for firefighting, increases its volume by about 300 times when exposed to a flame, forming a porous carbon layer that prevents oxygen from reaching the material’s surface and the spread of the flame.

To increase water resistance, a silane-based liquid hydrophobicizing solution, Beiphob FR (CHT Group, Tübingen, Germany), was used. These additives form a hydrophobic film on the sample, preventing water penetration and reducing capillary absorption.

### 2.2. Preparation of Engineered Wood Composite

The biopolyurethane binder was prepared from polyol (43.25%), isocyanate (35%) and rapeseed oil (21.75%). A specified amount of rapeseed oil was added to the required amount of polyol, and the mixture was mixed with an electric mixer Bosch MFQ 3010 (Gerlingen-Schillerhöhe, Ludwigsburg, Germany) for 30 s at 1300 rpm. The calculated amount of isocyanate was added to the mixture, and it was mixed again for 60 s. When an expandable graphite additive was used, it was added to the polyol and mixed in it before preparing the biopolyurethane binder. The prepared binder was poured onto the prepared straws, and the moulding mixture was thoroughly mixed with a mobile mixer for 60 s. The prepared moulding mixture was placed in a mould and compressed using a hydraulic press Tongrun T40 (Shanghai Tongrun Imp. & Exp. Co., Ltd., Shanghai, China) at a specified rate of 100 mm/min.

When using a hydrophobizer, after mixing it in water at a temperature of (23 ± 5 °C), a 10% concentration solution was prepared, in which the straw was soaked for 24 h. After immersion, the straw was drained, placed on shelves in a 30 mm thick layer, and dried in a drying oven until a constant weight was reached.

The composites were formed in a 300 × 100 × 100 mm metal mould, maintaining the set compression level for 20 min. Samples for property determination tests were prepared from the formed composites with a band saw. Composites were formed from each different fraction of straw, using different filler and binder ratios (1:0.5; 1:0.75; 1:1; 1:1.25; 1:1.5) and different compression levels (1.5 MPa, 2.25 MPa, 3 MPa).

Before testing, the samples were stored for 72 h at a temperature of 23 ± 2 °C and 50 ± 5% relative humidity.

**Figure 1:**
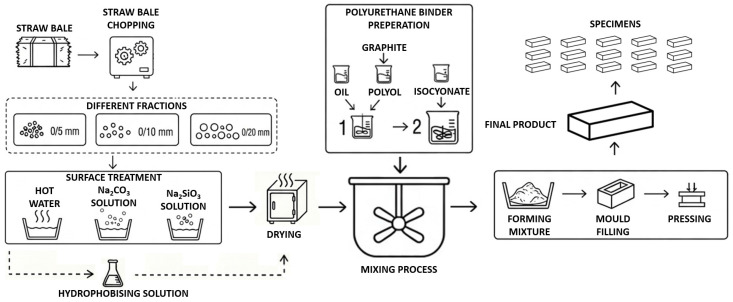
General scheme showing the preparation of engineered wood composite specimens.

The general scheme of composite preparation is presented in Figure 1.

### 2.3. Test Methods

#### 2.3.1. Straw Particle Studies

The bulk density of straw of different fractions was determined using a metal container of known volume, scales and a ruler. Straw was poured into the metal container from the same height (about 20–30 cm above the container), its excess was levelled with a ruler, and the container with the straw sample was weighed. The bulk density of the sample was calculated according to Formula (1):

(1) $$\rho =\frac{m}{V},{kg/m}^{3},$$

where *ρ*—bulk density of the sample, kg/m^3^; *m*—mass of the sample, kg; and *V*—volume of the container, m^3^. To obtain one result, 5 samples from each fraction were measured.

A set of sieves with mesh sizes of 20, 10, 5, 2.5, 1.25, 0.63, and 0 mm (bottom) was used to determine the granulometric composition of straw fractions. A 100 g sample poured into a sieve column was manually sieved for 60 s. After sieving, the mass of straw remaining on each sieve was weighed; the residue on each sieve, the total residue, and the weight passing through each sieve were calculated and expressed as percentages.

The density of straw of different fractions under different loads was determined using metal cylinders with a diameter of 205 mm and a height of 110 mm, placed on metal plates measuring 300 × 300 × 10 mm and needle-type metal pressure gauges providing loads of 50 Pa, 100 Pa, 250 Pa, 500 Pa, 1000 Pa and 2000 Pa. The straw was compressed under different loads with the pressure gauges for 2 min, and its height was then measured. The density of straw was calculated according to Formula (2):

(2) $$\rho_{0}=\frac{m}{0.785\cdot D^{2}\cdot h},\frac{kg}{m^{3}},$$

where *ρ*_0_—density of the straw sample, kg/m^3^; *m*—mass of the straw sample, kg; *D*—diameter of the metal cylinder, m; and *h*—height of the straw sample compressed in the metal cylinder, m. To obtain one result, 3 samples from each fraction were sieved.

The shape of straw particles was analysed using a public-domain software programme, ImageJ 1.54. For this purpose, straw milled through sieves of different sizes was sieved through a sieve column, and representative samples were taken from each sieve. The samples were photographed and transferred to the software. The particle aspect ratio, roundness, and circularity were evaluated during the analysis. The aspect ratio is the ratio of the particle’s width to its height. Roundness is defined as the geometry of an object approximated as a perfect circle. Circularity quantifies the degree to which a shape is round.

#### 2.3.2. Research on the Properties of Engineered Wood Composite

Composite compressive stress tests were performed based on the methodology specified in the test standard EN ISO 29469:2023 [49], using a computer-controlled testing machine HOUNSFIELD H10KS (Hounsfield Ltd., Salfords, UK). To obtain a single result, 3 samples measuring 20 × 20 × 20 mm were cut from each composite composition.

The thickness swelling tests of the composites after immersion in water were performed in accordance with the methodology specified in the test standard EN 317:1999 [50]. The specimens were immersed vertically in water with the forming surface so that the upper surface was covered by 25 ± 5 mm and kept in water for 24 h. The result of the thickness swelling after immersion in water was calculated according to Formula (3):

(3) $$G_{t}=\frac{t_{2}-t_{1}}{t_{1}}\cdot 100,\%,$$

where *G_t_*—thickness swelling after immersion in water, %; *t*_1_—sample thickness before immersion, mm; and *t*_2_—sample thickness after immersion, mm.

To obtain one result, 3 samples were cut from each composite composition, each measuring 50 × 50 × 10 mm.

The short-term water absorption tests on the composites were carried out in accordance with the methodology specified in the test standard EN ISO 29767:2019 [51]. The samples were immersed in water so that their lower surfaces were covered by 10 ± 2 mm of water and were kept for 24 h. After immersion, excess water was drained from the samples, and they were weighed. The result of short-term water absorption was calculated according to Formula (4):

(4) $$W_{p}=\frac{m_{24}-m_{0}}{A_{p}},kg/m^{2},$$

where *W_p_*—short-term water absorption, kg/m^2^; *m*_24_—mass of the partially submerged sample after 24 h of exposure, kg; *m*_0_—initial mass of the dry sample, kg; and *A_p_*—area of the sample’s submerged surface, m^2^. To obtain a single result, 3 samples measuring 50 × 50 × 15 mm were cut from each composite.

Combustion tests of the composites were performed based on the methodology specified in the test standard EN 11925-2:2010 [52]. The samples were exposed to flame for 15 s and 30 s. After the specified duration of exposure to flame, the height of the flame, whether the flame reached a height of 150 mm, and whether combustion was maintained after removing the flame source were recorded. To obtain one result, 3 samples of 250 × 90 × 10 mm in size were cut from each composition of the composites.

The structural changes in the straw and composite were evaluated using scanning microscopy. For this purpose, a JEOL JSM-7600F scanning microscope (JEOL, Tokyo, Japan) was used.

To process the experimental data and evaluate their reliability, mathematical–statistical methods and the software STATISTICA v.8 were used.

## 3. Test Results and Discussion

**Figure 2:**
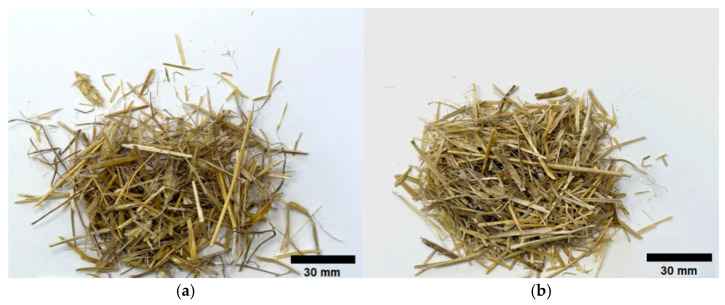

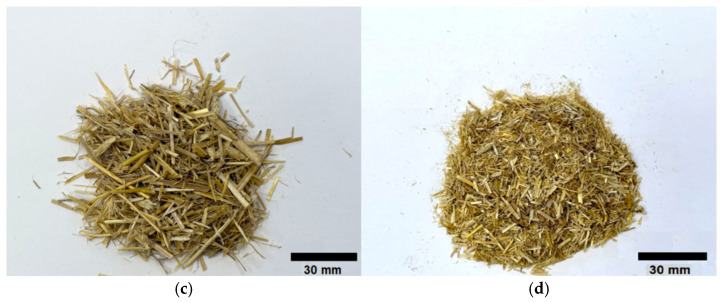
General view of straw particles after being milled through sieves with different mesh sizes, mm: (**a**) 35; (**b**) 25; (**c**) 10; (**d**) 5.

Pictures of straw milled through sieves of different sizes are shown in Figure 2. The figure shows not only the different-sized straw particles but also the different amounts of voids. The longer the straw, the greater the number of voids observed between the straw particles.

**Figure 3:**
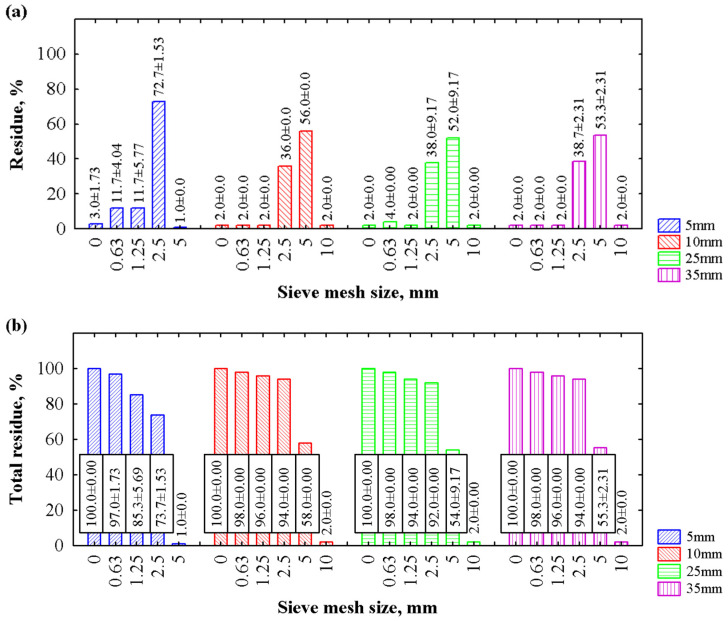

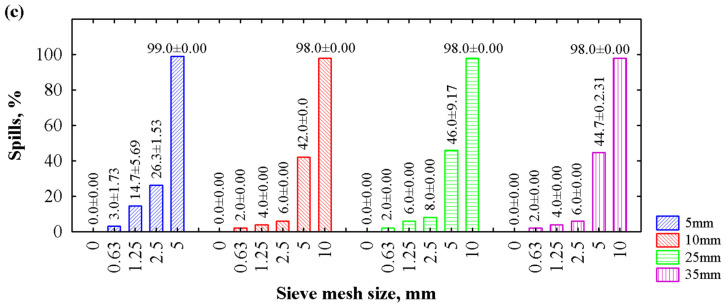
Distribution of the granulometric composition of straw particles when milling through sieves of different mesh sizes: (**a**) residue on the sieve, %; (**b**) total residue on the sieve, %; (**c**) passes, %.

The particle size distribution of straw milled through sieves with mesh sizes of 35, 25, 10 and 5 mm is shown in Figure 3.

As shown in Figure 3a, only when grinding through 5 mm mesh sieves does the dominant fraction change. If, when milling straw through 10, 25 and 35 mm mesh sieves, the dominant fraction was 5 mm and in all cases amounted to at least 52%, and the 2.5 mm fraction amounted to only about 38%, then when milling through 5 mm mesh sieves, the share of the 2.5 mm fraction exceeded 72%. Thus, the 2.5 mm fraction is formed at approximately 1.9 times the amount when milling through 10, 25, and 35 mm mesh sieves. Due to the reduced mesh size of the sieve, a larger amount of finer fraction straw was also formed. If the amount of straw in the 0.063 and 1.25 mm fractions was 2–4% when milling through larger sieves, then when milling through a sieve with a mesh size of 2.5 mm, the amount of straw in the 0.063 and 1.25 mm fractions was 11.7%. Meanwhile, the amount of fine particles formed on the bottom of the sieves changed very slightly, increasing from 2 to 3%.

**Figure 4:**
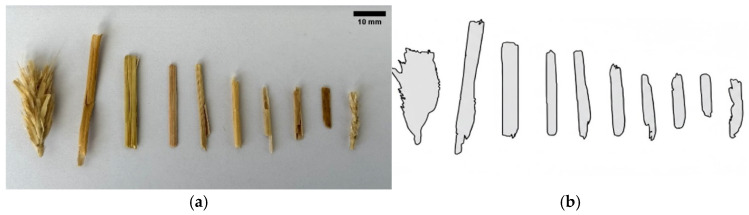
General view of straw after selection: (**a**) photographed; (**b**) processed by a program.

The shape of the straw ground and sieved through sieves of different mesh sizes was analysed according to three criteria: aspect ratio, roughness, and circularity. Initially, the selected straw particles were photographed (see Figure 4a); later, the program scanned their contours (see Figure 4b) and evaluated the particle shapes. The results for aspect ratio, roughness, and circularity are presented in Table 1.

**Figure 5:**
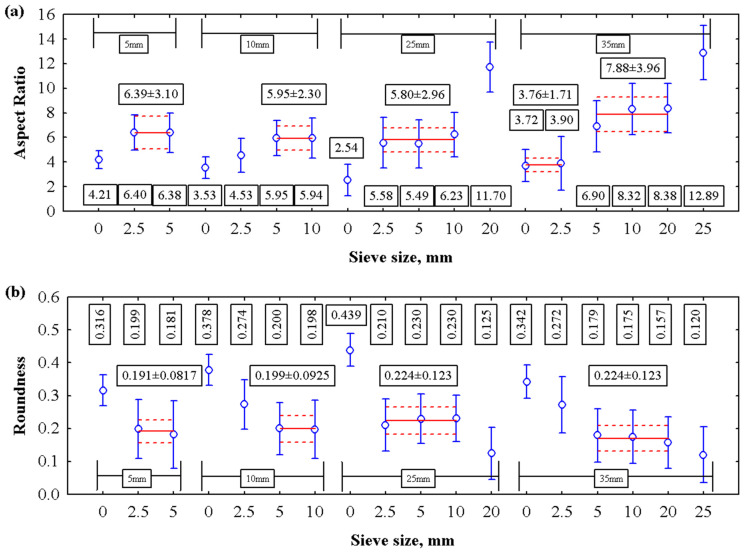

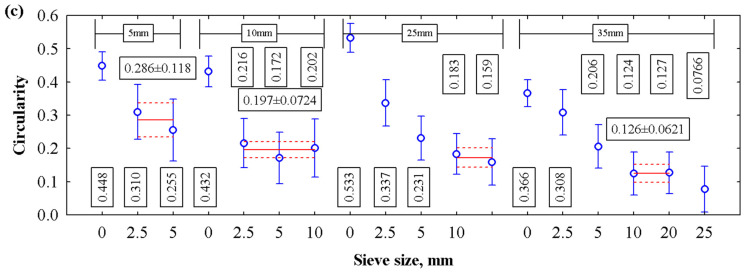
Straw particle geometry: (**a**)—particle aspect ratio; (**b**)—particle roundness; (**c**)—particle circularity (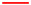
—mean value, 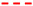
—confidence intervals).

The results obtained were processed statistically. The particle aspect ratio is shown graphically in Figure 5a. A statistical analysis of the aspect ratio properties of the 5 mm fraction residue on sieves with sizes of 0, 2.5, and 5 mm showed that the aspect ratio *p*-value was <0.05 (F = 5.68, 0.0052). This is a statistically significant difference between the average aspect ratios of at least one “sieve size” group. This means that the sieve size affects the aspect ratio. The residue values on the sieves were 0–4.21 ± 2.22, 2.5–6.40 ± 3.57, and 5–6.38 ± 2.55. A repeated analysis showed no difference in the aspect ratio between the 2.5 and 5 mm sieves (F = 0.000361, *p* = 0.99). The aspect ratio of 2.5 and 5 mm can be accepted as 6.39 ± 3.10.

A statistical analysis of the aspect ratio properties of the 10 mm fraction residue on sieves with sizes 0, 2.5, 5 and 10 mm showed that the aspect ratio *p*-value was <0.05 (F = 4.03, 0.010). This is a statistically significant difference between the average aspect ratios of at least one “sieve size” group. This means that the sieve size influences the aspect ratio. The residue values on the sieves were 0–3.53 ± 2.82, 2.5–4.53 ± 2.27, 5–5.95 ± 2.49, and 10–5.94 ± 2.17. A repeated analysis showed no difference in the aspect ratio between the 5 and 10 mm sieves; *p* > 0.05 (F = 0, *p* = 0.99). The aspect ratio of 5 and 10 mm can be accepted as 5.95 ± 2.30.

A statistical analysis of the aspect ratio properties of the 25 mm fraction residue on sieves with sizes 0, 2.5, 5, 10 and 20 mm was performed, which showed that the aspect ratio *p*-value was <0.05 (F = 14.58, 0.0). This is a statistically significant difference between the average aspect ratios of at least one “sieve size” group. This means that sieve size influences the aspect ratio. The residue values on the sieves were 0–2.54 ± 0.842, 2.5–5.58 ± 2.42, 5–5.49 ± 2.84, 10–6.23 ± 3.54, and 20–11.71 ± 7.03. The repeated analysis showed no difference in the aspect ratio between the 5 and 10 mm sieves (F = 0.24, *p* = 0.79). The aspect ratios of 2.5, 5 and 10 mm can be accepted as 5.80 ± 2.96.

The statistical analysis of the aspect ratio properties of the 35 mm fraction residue on sieves with sizes 0, 2.5, 5, 10, 20 and 25 mm showed that the aspect ratio *p*-value was <0.05 (F = 12.84, 0.0). This is a statistically significant difference in the aspect ratio averages of at least one “sieve size” group. This means that the sieve size affects the aspect ratio. The residue values on the sieves were 0–3.72 ± 1.91, 2.5–3.90 ± 1.03, 5–6.90 ± 3.69, 10–8.32 ± 4.47, 20–8.38 ± 3.89, and 25–12.89 ± 5.99. The repeated analysis showed no difference in the aspect ratio between the 0 and 2.5 mm sieves (F = 0.078, *p* = 0.789). The aspect ratios of 0 and 2.5 mm can be accepted as 3.76 ± 1.71. There is no difference in the aspect ratio between the 5, 10 and 20 mm sieves; *p* > 0.05 (F = 0.49, *p* = 0.62). The aspect ratios of 5, 10 and 20 mm can be accepted as 7.88 ± 3.96.

The analysis of particle roundness is presented in Figure 5b. A statistical analysis of the roundness properties of the 5 mm fraction residue on sieves with sizes 0, 2.5 and 5 mm showed that the *p*-value for the aspect ratio was <0.05 (F = 4.62, 0.013). This is a statistically significant difference between the roundness averages of at least one “sieve size” group. This means that the sieve size affects roundness. The residue values on the sieves were 0–0.316 ± 0.187, 2.5–0.199 ± 0.0906, and 5–0.181 ± 0.0718. A repeated analysis showed no difference in roundness between the 2.5 mm and 5 mm sieves (F = 0.248, *p* = 0.62). The roundness of particles 2.5 and 5mm can be accepted as 0.191 ± 0.0817.

A statistical analysis of the roundness properties of the 10 mm fraction residue on sieves with sizes 0–2.5–5–10 mm showed that the *p*-value for the aspect ratio was <0.05 (F = 7.78, 0.0). This is a statistically significant difference between the roundness averages of at least one “sieve size” group. This means that the sieve size affects roundness. The residue values on the sieves were 0–0.378 ± 0.167, 2.5–0.274 ± 0.129, 5–0.200 ± 0.0889, and 5–0.198 ± 0.102. The repeated analysis showed no difference in roundness between the 5 and 10 mm sieves (F = 0.002, *p* = 0.96). The roundness of particles 2.5 and 5mm can be accepted as 0.199 ± 0.0925.

A statistical analysis of the roundness properties of the 25 mm fraction residue on sieves with sizes 0, 2.5, 5, 10, and 20 mm showed that the *p*-value for the aspect ratio was <0.05 (F = 15.6, 0.0). This is a statistically significant difference between the average roundness of at least one “sieve size” group. This means that the sieve size affects the roundness. The residue values on the sieves were 0–0.439 ± 0.150, 2.5–0.210 ± 0.0854, 5–0.230 ± 0.111, 10–0.230 ± 0.160, and 20–0.125 ± 0.0901. The repeated analysis showed no difference in roundness between the 5 and 10 mm sieves (F = 0.092, *p* = 0.91). The roundness of particles 2.5, 5 and 10 mm can be accepted as 0.224 ± 0.123.

The statistical analysis of the roundness properties of the 35 mm fraction residue on sieves with sizes 0, 2.5, 5, 10, 20, and 25 mm showed that the aspect ratio *p*-value was <0.05 (F = 6.82, 0.0). This is a statistically significant difference between the average roundness of at least one “sieve size” group. This means that the sieve size affects the roundness. The residue values on the sieves were 0–0.342 ± 0.173, 2.5–0.272 ± 0.0690, 5–0.179 ± 0.0779, 10–0.175 ± 0.152, 20–0.157 ± 0.102, and 25–0.120 ± 0.121. The repeated analysis showed no difference in roundness between the 5 and 10 mm sieves (F = 0.12, *p* = 0.89). The roundness of particles 5, 10, 20 mm can be accepted as 0.170 ± 0.111.

A graphical interpretation of the results of the straw particle circularity analysis is presented in Figure 5c. A statistical analysis of the properties of the 5 mm fraction residue on sieves with sizes 0, 2.5 and 5 mm circularity showed that the *p*-value of the circularity is <0.05 (F = 9.73, 0.0). This is a statistically significant difference between the circularity means of at least one “sieve size” group. This means that the sieve size affects the circularity. The residue values on the sieves were 0–0.448 ± 0.160, 2.5–0.310 ± 0.135, and 5–0.255 ± 0.0893. A repeated analysis showed no difference in the circularity between the 2.5 and 5mm sieves; *p* > 0.05 (F = 1.22, *p* = 0.28). The circularity of 2.5 and 5 mm can be accepted as 0.286 ± 0.118.

A statistical analysis of the circularity properties of the 10 mm fraction residue on sieves with sizes 0, 2.5, 5 and 10 mm showed that the circularity *p*-value was <0.05 (F = 17.6, 0.0). This is a statistically significant difference between the circularity means of at least one “sieve size” group. This means that the sieve size affects the circularity. The residue values on the sieves were 0–0.432 ± 0.182, 2.5–0.216 ± 0.0454, 5–0.172 ± 0.0902, and 10–0.202 ± 0.0751. A repeated analysis showed no difference in circularity among the 2.5, 5 and 10 mm sieves (F = 1.33, *p* = 0.28). The circularity of 2.5, 5, and 10 mm can be accepted as 0.197 ± 0.0724.

The statistical analysis of the properties of the 25 mm fraction residue on sieves with sizes 0, 2.5, 5, 10, and 20 mm was performed, and the circularity *p*-value was <0.05 (F = 35.6, 0.0). This is a statistically significant difference between the circularity means of at least one “sieve size” group. This means that the sieve size affects the circularity. The residue values on the sieves were 0–0.533 ± 0.140, 2.5–0.337 ± 0.127, 5–0.231 ± 0.107, 10–0.183 ± 0.0674, and 20–0.159 ± 0.0773. The repeated analysis showed no difference in the circularity of the 10 and 20 mm sieves (F = 0.69, *p* = 0.42). The circularity of 10 mm and 20 mm can be accepted as 0.173 ± 0.0714.

The statistical analysis of the properties of the 35 mm fraction residue on sieves with sizes 0, 2.5, 5, 10, 20 and 25 mm showed that the circularity *p*-value was <0.05 (F = 18.2, 0.0). This is a statistically significant difference between the circularity means of at least one “sieve size” group. This means that the sieve size affects the circularity. The residue values on the sieves were 0–0.366 ± 0.146, 2.5–0.308 ± 0.132, 5–0.206 ± 0.0734, 10–0.124 ± 0.0641, 20–0.127 ± 0.0630, and 20–0.0766 ± 0.0439. The repeated analysis showed no difference in circularity between the 10 and 20 mm sieves (*p* > 0.05; F = 0.0086, *p* = 0.93). The circularity of 10 mm and 20 mm can be accepted as 0.126 ± 0.0621.

**Figure 6:**
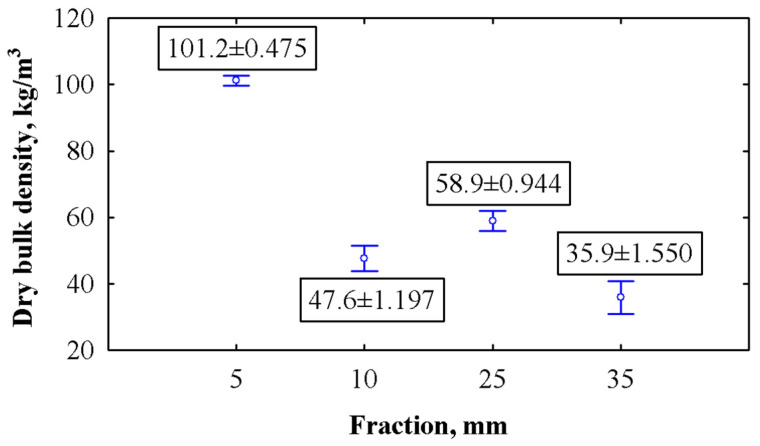
Graphical interpretation of the bulk density of straw particles of different fractions.

The influence of different-sized straw particles on the bulk density of straw was further evaluated. Straw milled through a 5 mm mesh sieve has a significantly higher density than straw milled through a larger mesh sieve. The images of straw in Figure 2 showed that the elimination of even a small amount of longer straw particles ensures better convergence of straw particles. Conversely, even a small amount of longer straw particles leads to the formation of large gaps between straw particles. Experimental studies of the bulk density of straw particles showed that it depends on the fraction size, which ranged from 5 to 35 mm (Figure 6). Statistical analysis showed a significant difference in the bulk density of straw particles across fraction sizes. The statistical analysis data are presented in Table 1. With a straw particle fraction of 5 mm, their bulk density was 101.2 ± 0.475 kg/m^3^. With an increase in the particle fraction from 5 to 10 mm, the bulk density was 47.6 ± 1.197 kg/m^3^. It was found that increasing the particle fraction from 5 to 10 mm decreased the bulk density by ~53.0%. With a 25 mm straw particle fraction, their bulk density was 58.9 ± 0.944 kg/m^3^. We determined that as the fraction increased from 10 to 25 mm, the bulk density increased by ~23.7%. With a straw fraction of 35 mm, their bulk density was 35.9 ± 1.550 kg/m^3^. With an increase in the straw fraction from 25 to 35 mm, the bulk density decreased by ~39.1%.

**Figure 7:**
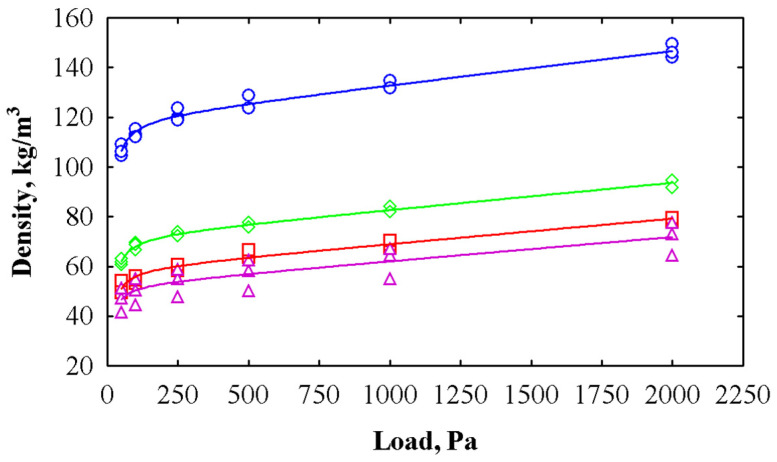
Dependence of the density of straw of different fractions (5 to 35 mm) on different loads: ○—5 mm fraction; □—10 mm fraction; ◊—25 mm fraction; ∆—35 mm fraction.

After milling the straw through sieves of different mesh sizes, particles of varying sizes were obtained, each with varying resistance to loads. Figure 7 presents the changes in the density of milled straw particles of different fractions under different loads.

Figure 7 shows the dependence of the density of straw particles of different fractions (5 to 35 mm) on the load, which ranged from 50 to 2000 Pa. It was found that the lowest density was obtained for straw particles of the 35 mm fraction, with the load varying from 50 to 2000 Pa, and the density changed from 46.7 ± 4.974 kg/m^3^ to 71.7 ± 6.691 kg/m^3^. For straw particles of the 10 mm fraction, the density changed from 51.6 ± 2.406 kg/m^3^ to 79.0 ± 1.155 kg/m^3^; the density of the 25 mm fraction changed from 61.8 ± 1.250 kg/m^3^ to 93.7 ± 1.674 kg/m^3^. The highest density was obtained for the 5mm fraction straw pellets, which ranged from 107 ± 2.371 kg/m^3^ to 147 ± 2.706 kg/m^3^. Experimental data analysis showed that the largest increase in density occurred from 50 to 500 Pa.

According to the experimental data, the relationship between the density of straw particles of different fractions and the loading level was described by equations (Table 2).

**Figure 8:**
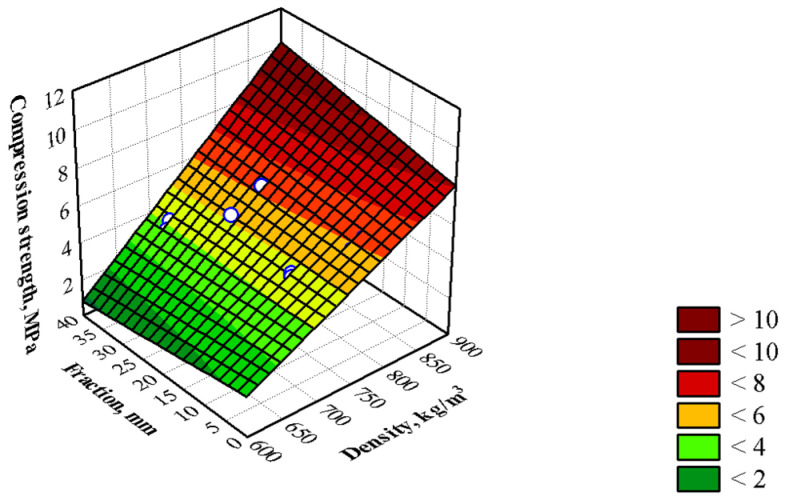
Compressive stress dependence on density and fraction size, ○—average of composition results.

After forming samples from straw and polyurethane adhesive, the influence of straw density and fraction size on the compressive strength of the engineered wood composite was evaluated. Figure 8 shows the dependence of compressive stress on the material’s density and fraction. When producing a composite from straw particles, the binder content was set to 0.75, and the compression level was 2.25 MPa. Experimental studies showed that the compressive stress in the composite increases linearly with the material’s density. As the density of the composite increased from 697.2 to 820.3 kg/m^3^, the compressive stress increased from 3.84 to 6.60 MPa, representing a ~72% increase. Meanwhile, the influence of the fraction on the compressive stress showed that as the fraction size increased from 5 to 35 mm, the compressive stress decreased from 6.26 ± 0.340 to 4.10 ± 0.234 MPa, a ~12% decrease. The material’s density exerts the greatest influence on compressive stress. According to experimental data, the relationship between the compressive stress of the composite and the material density and fraction size was described by Equation (9), and the graphical interpretation is presented in Figure 8:

(9) $$\sigma_{10\%}=-9.5453+0.019534\cdot \rho -0.214380\cdot fraction+0.0002994\cdot \rho \cdot fraction$$

with standard deviation Sr = 0.442 MPa (n = 12), regression coefficient R = 0.918 and determination coefficient R^2^ = 0.9843.

**Figure 9:**
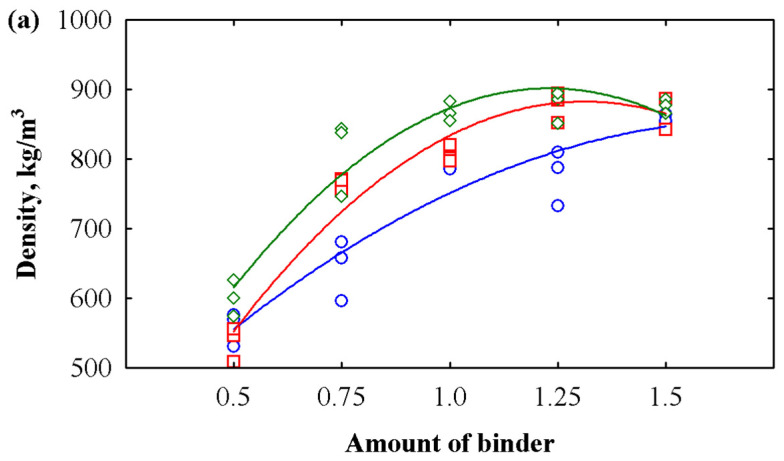

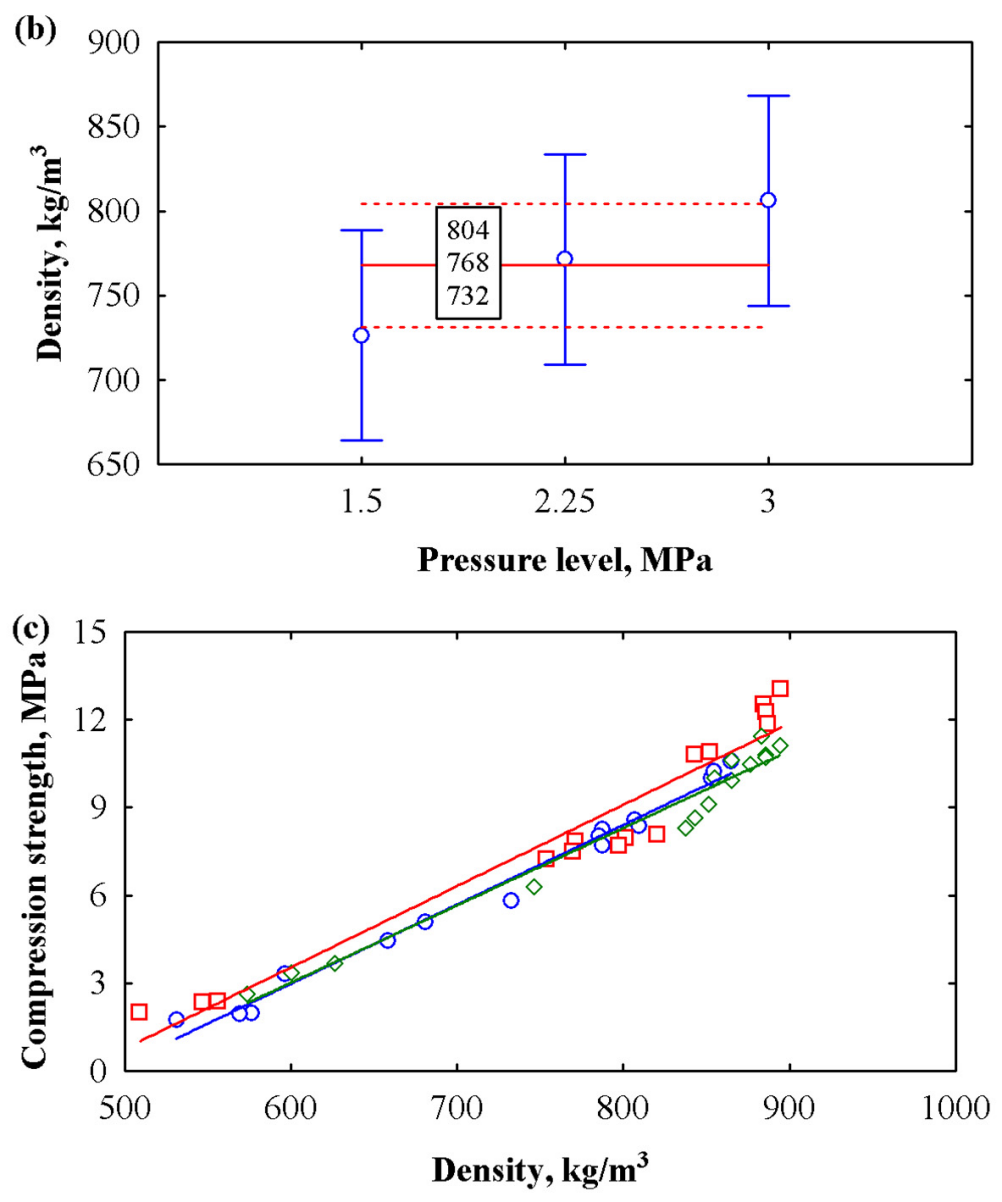
Compression studies of engineered wood composites made of straw particles: (**a**)—relationship between composite density and binder content ○—when pressure is 1.5 MPa, □—2.25 MPa, and ◊—3.0 MPa; (**b**)—influence of pressure on composite density, (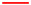
—mean value, 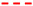
—confidence intervals, ○—average of composition results.); (**c**)—relationship between compressive stress and material density.

Since the highest strength was achieved using the 5 mm fraction, 5 mm straw particles were selected for further studies of the engineered wood composite. For compression tests, specimens were formed with a binder content ranging from 0.5 to 1.5, and the pressure level ranged from 1.5 to 3.0 MPa. First, the relationship between the composite density and the binder content was evaluated (see Figure 9a).

**Figure 10:**
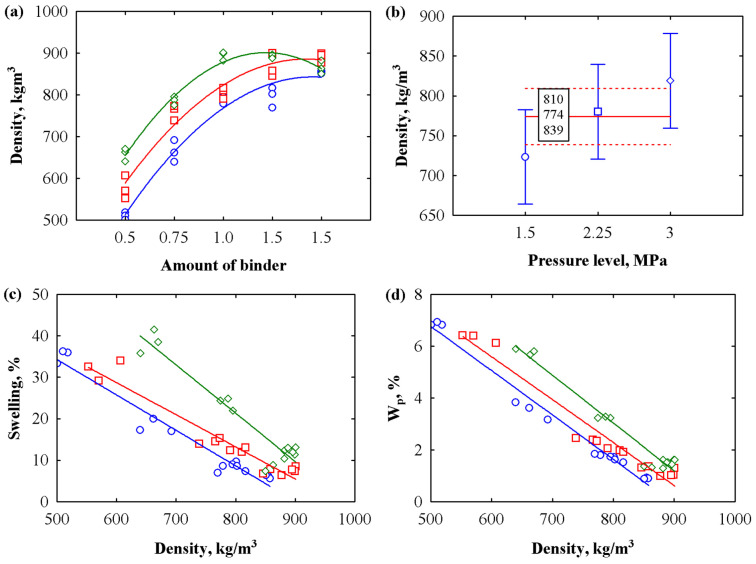
Studies of the moisture properties of a composite made of straw particles: (**a**)—the relationship between the density of the composite and the amount of binder ○—when the pressure is 1.5 MPa, □—2.25 MPa, and ◊—3.0 MPa; (**b**)—the influence of pressure level on the density of the composite, (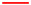
—mean value, 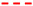
—confidence intervals, ○—average of composition results); (**c**)—the relationship between swelling and the density of the material ○—when the pressure is 1.5 MPa, □—2.25 MPa, and ◊—3.0 MPa; (**d**)—the relationship between water absorption and the density of the material ○—when the pressure is 1.5 MPa, □—2.25 MPa, and ◊—3.0 MPa.

Experimental studies showed that when increasing the binder content from 0.5 to 1.5, the increase in density at a pressure of 1.5 MPa varied from 600 ± 26.3 to 876 ± 10.0 kg/m^3^, at a pressure level of 2.25 MPa, it varied from 559 ± 24.4 to 857 ± 6.31 kg/m^3^, and at a pressure of 3.0 MPa, it varied from 537 ± 24.9 to 872 ± 25.3 kg/m^3^. The graph shows that the greatest increase in density occurred from 0.5 to 1.0, and thereafter, the density increased relatively little. Also, the relationship between the composite density and the binder content was described by an equation (Table 3), and the graphical interpretation is presented in Figure 10a.

Next, an analysis of the composite density as a function of the pressure level was performed (Figure 9b), which showed that the density of the composites is not strongly dependent on the pressure level. Statistical analysis showed an insignificant difference between the studies’ results: F = 1.689; *p* = 0.197; R = 0.273; R^2^ = 0.0744; 768 ± 121 kg/m^3^. At a pressure level of 1.5 MPa, the composite density was 726 ± 115 kg/m^3^, at 2.25 MPa, it was 771 ± 130 kg/m^3^, and at 3.0 MPa, it was 806 ± 113 kg/m^3^.

After performing compression tests and evaluating the effects of binder content and pressure on the material’s density, an attempt was made to assess the effects of compressive stress on the material’s density. According to the experimental data, the relationship between the composite’s compressive stress and material density was described by equations (Table 3), and the graphical interpretation is presented in Figure 9c. The analysis showed that the compressive stress varies linearly with increasing material density. The graph shows that at pressure levels of 1.5 MPa and 3.0 MPa, the compressive stress was the same. Meanwhile, at 2.25 MPa, the compressive stress was higher than at 1.5 MPa and 3.0 MPa. Assuming a material density of 700 kg/m^3^, the compressive stress at 1.5 MPa was 5.7 MPa, at 2.25 MPa, it was 6.3 MPa, and at 3.0 MPa, it was 5.7 MPa. The difference in compressive stress between 1.5, 3.0, and 2.25 was ~11%.

Figure 10 presents moisture property studies on untreated 5 mm straw particles, varying the binder content from 0.5 to 1.5 and the pressure level from 1.5 to 3.0 MPa.

Based on the experimental data, we first evaluated the relationship between the composite density and the binder content (see Figure 10a). We determined that with an increase in the amount of binder from 0.5 to 1.5, the increase in density at a pressure of 1.5 MPa changed from 510 ± 8.78 to 854 ± 3.37 kg/m^3^, at a pressure of 2.25 MPa, it changed from 576 ± 27.7 to 891 ± 11.7 kg/m^3^, and at a pressure of 3.0 MPa, it changed from 658 ± 15.6 to 865 ± 15.7 kg/m^3^. It was found that the largest increase in density occurred between 0.5 and 1.0, after which the density increased relatively little. Also, the relationship between the composite density and the binder content was described by an equation (Table 4), and the graphical interpretation is presented in Figure 10a.

The analysis of composite density as a function of pressure (Figure 10b) showed that it varies little. Statistical analysis showed an insignificant difference between the test results: F = 2.663; *p* = 0.0815; R = 0.335; R^2^ = 0.113; 774 ± 118 kg/m^3^. At a pressure level of 1.5 MPa, the density of the composite was 723 ± 129 kg/m^3^, at 2.25 MPa, it was 780 ± 117 kg/m^3^, and at 3.0 MPa, it was 819 ± 93.3 kg/m^3^.

After conducting moisture properties studies and evaluating the effects of binder content and compression on the material’s density, the swelling and water absorption results were analysed as functions of density. According to the experimental data, the relationships between the swelling and water absorption of the composite and the material density were described by Equations (19)–(24) (Table 4), and the graphical interpretation is presented in Figure 10c,d.

**Figure 11:**
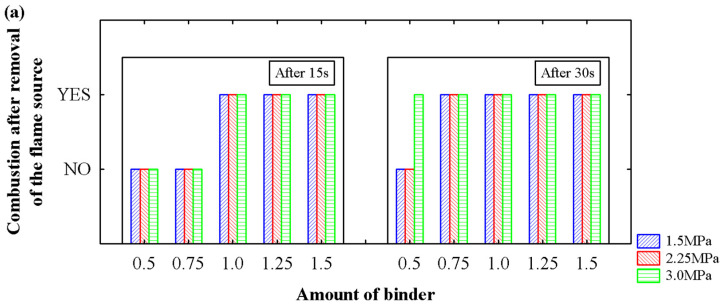

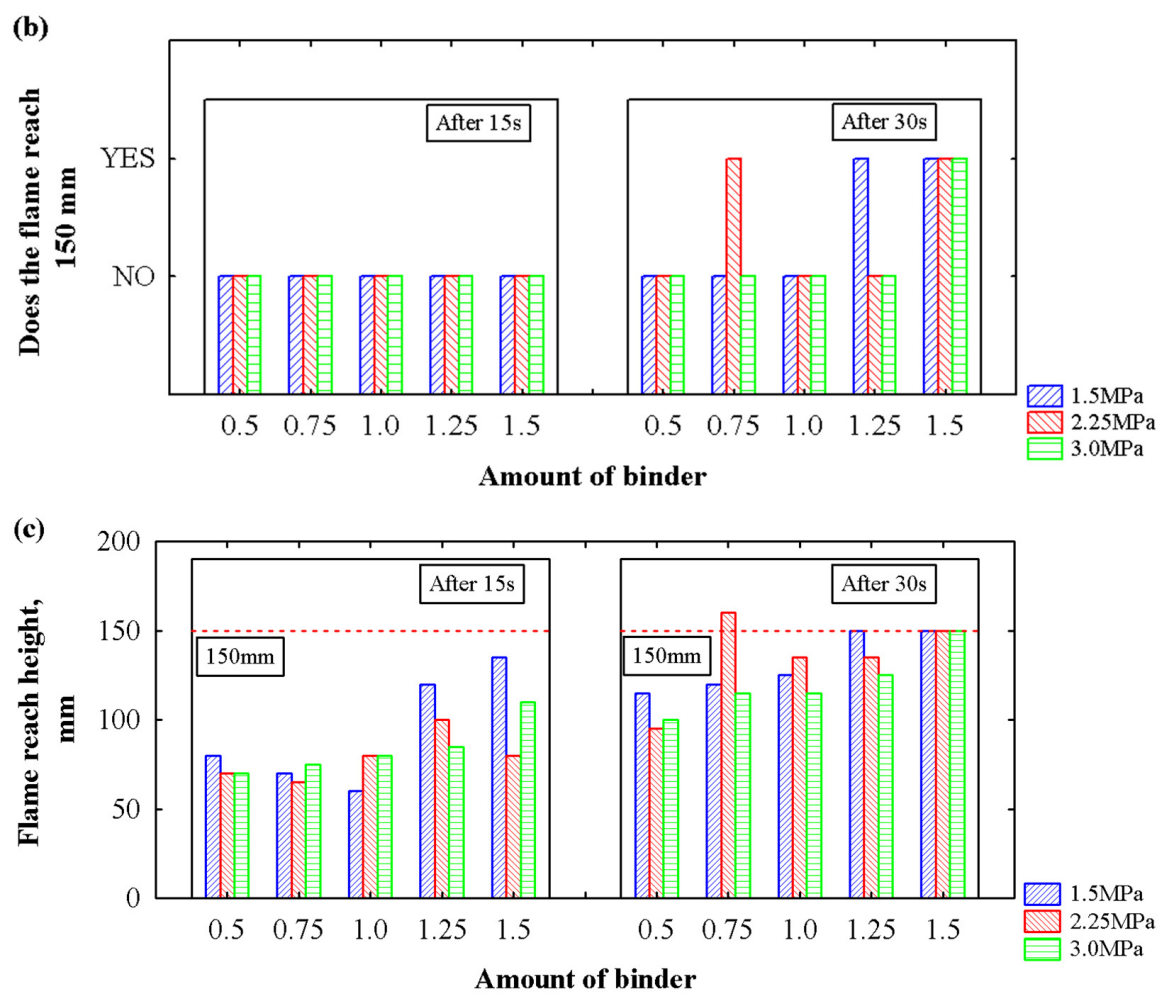
Flammability tests of a composite made of 5 mm untreated straw particles: (**a**)—combustion after removing the flame source; (**b**)—whether the flame reaches 150 mm; (**c**)—flame height, mm.

Figure 11 presents the flammability test results for the engineered wood composite. The evaluation assessed whether the sample burned after removing the flame source, whether the flame reached the 150 mm mark, and the maximum flame height on the sample surface. The tests showed that both the binder content and the compression level affected the sample’s flammability and flame spread. The flame spread across the sample surface when the largest amount of binder was used. Since the flame spread time was short, the flame spread height was an important indicator. The test results showed that with a binder/filler ratio of 0.5–1.0, the flame spread height did not exceed 100 mm in any case when the flame was applied for 15 s. When the flame was applied for 30 s and binder ratios of 0.5 and 1.0 were used, the flame spread height did not exceed 150 mm. Meanwhile, at a binder ratio of 0.75 and a compression level of 2.25 MPa, the flame propagation height exceeded the permissible 150 mm. This could be due to random factors such as material heterogeneity due to uneven binder distribution, the formation of air gaps, or random impurities.

**Figure 12:**
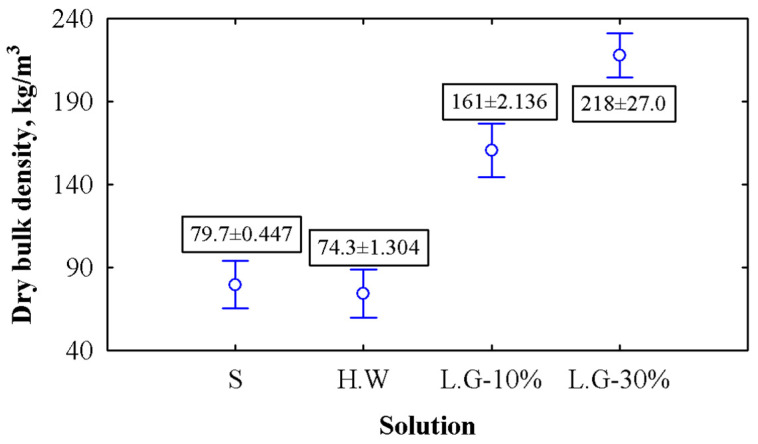
Influence of bulk density after treating 5 mm fraction straw particles with various solutions: S—sodium carbonate; H.W—hot water; L.G-10%—10% sodium silicate liquid glass; L.G-30%—30% sodium silicate liquid glass.

To improve the adhesive properties of the fillers and binder in the resulting composite, the straw was treated with different methods. During the treatment, the initial properties of the straw itself, such as density, granulometric composition and load resistance, changed first. After treating 5 mm straw in four different ways (sodium carbonate, hot water, 10% and 30% liquid glass solution), the bulk density was determined (see Figure 12). The analysis of the bulk density of 5 mm fraction straw particles showed that it depended on the treatment of particles with various solutions. Statistical analysis showed a significant difference in the results of the 5 mm fraction straw pellets, with analysis data: F = 112.5, *p* = 0, R = 0.977, and R^2^ = 0.955. The lowest bulk density observed for 5 mm fraction straw particles treated with hot water was 74.3 ± 1.304 kg/m^3^, and for those treated with sodium carbonate, it was 79.7 ± 0.447 kg/m^3^. The highest bulk density observed for 5 mm fraction straw particles treated with 30% liquid glass solution was 218 ± 27.0 kg/m^3^, and for those treated with 10% liquid glass solution, it was 161 ± 2.136 kg/m^3^. The largest difference in bulk density was observed for 5mm fraction straw particles treated with hot water and a 30% liquid glass solution, at 2.93 times.

**Figure 13:**
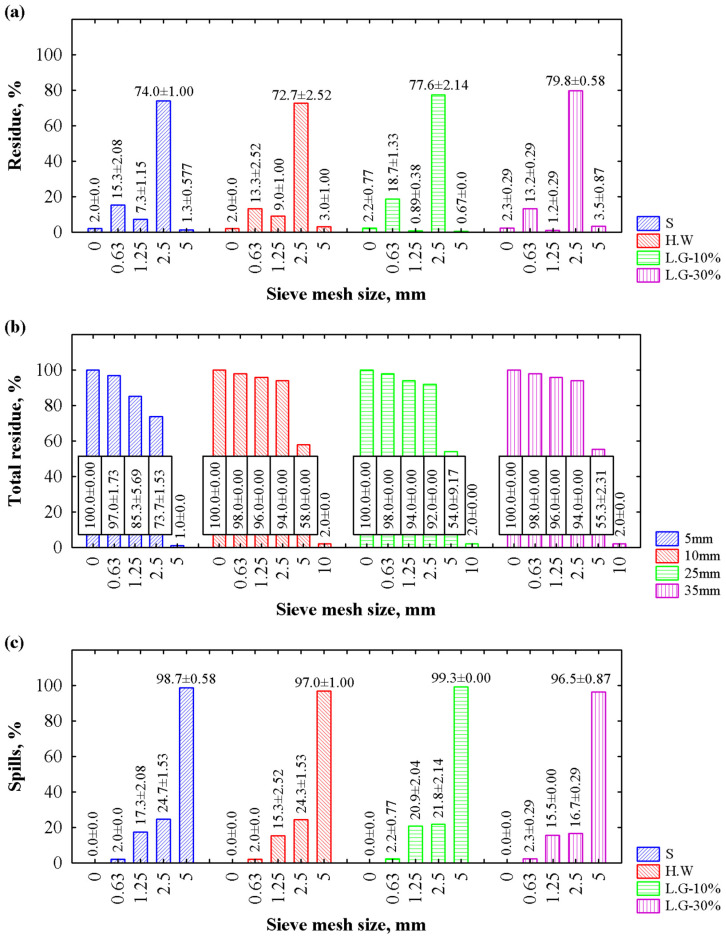
Granulometric composition of 5mm fraction straw particles treated with different solutions: (**a**)—sieve residue, %; (**b**)—total sieve residue, %; (**c**) passes, %; S—sodium carbonate; H.W—hot water; L.G-10%—10% liquid glass; L.G-30%—30% liquid glass.

Figure 13 shows the granulometric composition obtained from 5 mm fraction straw particles treated with four different solutions (sodium carbonate, hot water, 10% and 30% liquid glass solution).

**Figure 14:**
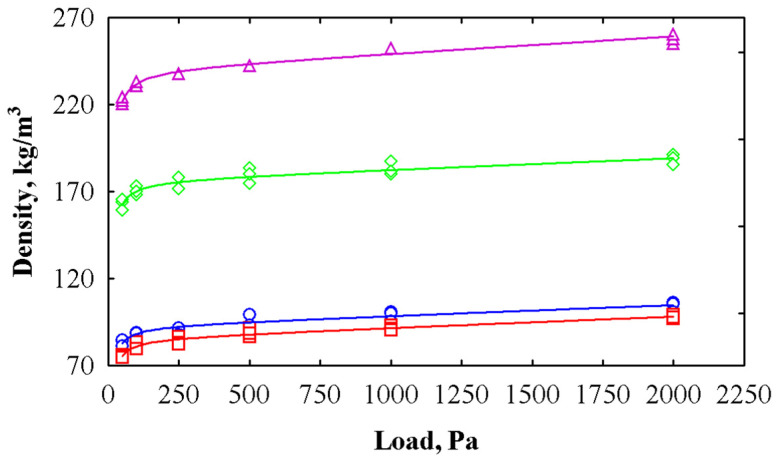
Density dependence of 5 mm fraction straw particles after treatment with various solutions on the load: ○—sodium carbonate; □ hot water; ◊—10% liquid glass; ∆—30% liquid glass.

Figure 14 shows the density dependence of 5mm fraction straw particles after treatment with various solutions on the load, which ranged from 50 to 2000 Pa (Figure 14). The lowest density was obtained after hot water treatment, with the load varying from 50 to 2000 Pa; the density changed from 75.4 ± 0.719 to 98.0 ± 1.216 kg/m^3^. After treatment with sodium carbonate, the density changed from 83.4 ± 1.825 to 104 ± 2.840 kg/m^3^. After treatment with 10% liquid glass solution, the density changed from 163 ± 3.012 to 189 ± 2.979 kg/m^3^. The highest density was obtained after processing straw particles with a 30% liquid glass solution, ranging from 222 ± 2.041 to 258 ± 2.744 kg/m^3^. Experimental data analysis showed that the largest increase in density occurred between 50 and 500 Pa.

According to the experimental data, the density relationship of 5 mm fraction straw particles after processing with various solutions was described by equations (Table 5), and the graphical interpretation is presented in Figure 14.

**Figure 15:**
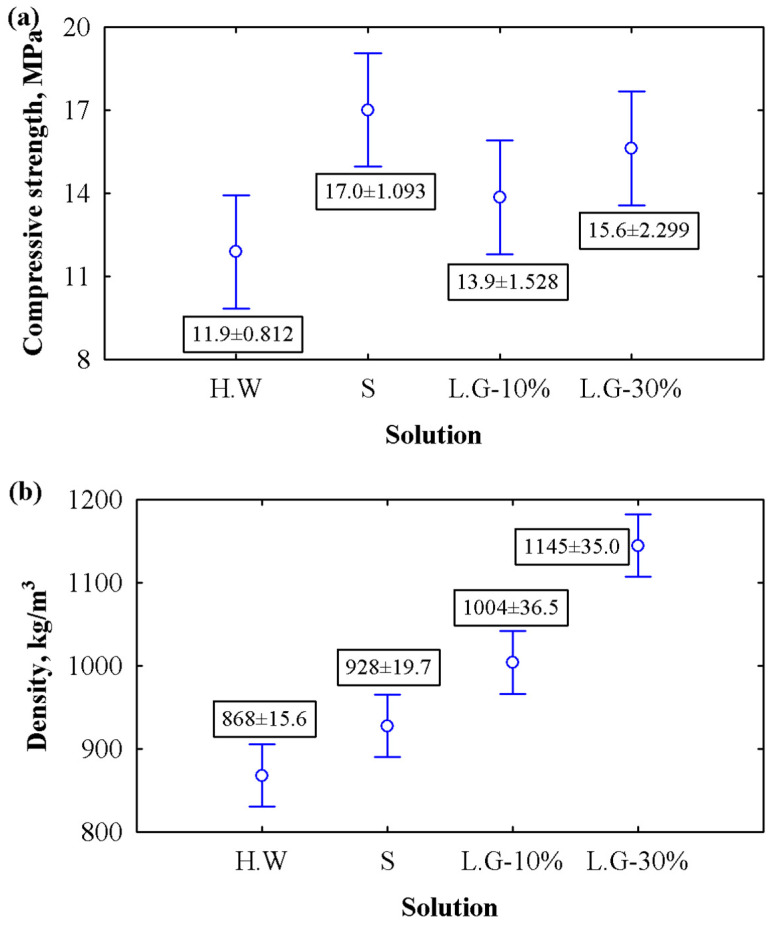
The influence of treatment on density and compressive strength of engineered wood composites: (**a**)—dependence on density; (**b**)—dependence on compressive strength; H.W—hot water; S—sodium carbonate; L.G-10%—10% liquid glass; L.G-30%—30% liquid glass.

Compression tests were performed after treating 5 mm straw particles with four different solutions (sodium carbonate, hot water, 10% and 30% liquid glass solution), and the compressive stress was determined (see Figure 15a). The analysis showed that the compressive stress depended on the treatment of straw particles with various solutions. Statistical analysis showed F = 6.24, *p* = 0.017, R = 0.837, and R^2^ = 0.701.

The analysis of the compressive stress of 5 mm fraction straw particles showed that the density depended on the particles’ treatments with various solutions (Figure 15b). Statistical analysis showed F = 53.7, *p* = 0.0, R = 0.976, and R^2^ = 0.953.

Since the *p*-value was <0.05, there was a statistically significant difference between the averages of compressive stresses and densities of at least one group of composites made from straw particles treated with different solutions. This means the type of solution affected compressive stress and density.

The 5 mm fraction straw particles treated with hot water had the lowest compressive stress (11.9 ± 0.812 MPa) and the lowest density (868 ± 15.6 kg/m^3^). The highest compressive stress was exhibited by the 5 mm fraction of straw particles treated with a sodium carbonate solution, at 17.0 ± 1.093 MPa, and the density was 928 ± 19.7 kg/m^3^. After processing straw with 10% liquid glass solution, the values were 13.9 ± 1.528 MPa—1004 ± 36.5 kg/m^3^, and after processing with 30% liquid glass solution, the values were 15.6 ± 2.299 MPa—1145 ± 35.0 kg/m^3^. The highest densities were observed for straw treated with 10–30% liquid glass solutions.

Comparing samples prepared from straw treated with hot water and treated with 30% liquid glass solution, we observed a ~32% difference in density and a ~31% difference in compressive stress. Meanwhile, comparing the samples prepared from straw treated with hot water and a sodium carbonate solution, we determined that the density difference was ~7%, and the compressive stress difference was ~43%. When comparing samples prepared from straw treated with sodium carbonate and a 30% liquid glass solution, the density difference was ~23%, and the compressive stress difference was ~9%.

It was determined that after treating straw with liquid glass solutions, the density of the resulting samples increased significantly compared to that of the samples treated with a sodium carbonate solution, while the compressive stress was lower. The greatest influence on the compressive stress of the samples was exerted by the 5 mm fraction of sodium carbonate-treated straw particles.

**Figure 16:**
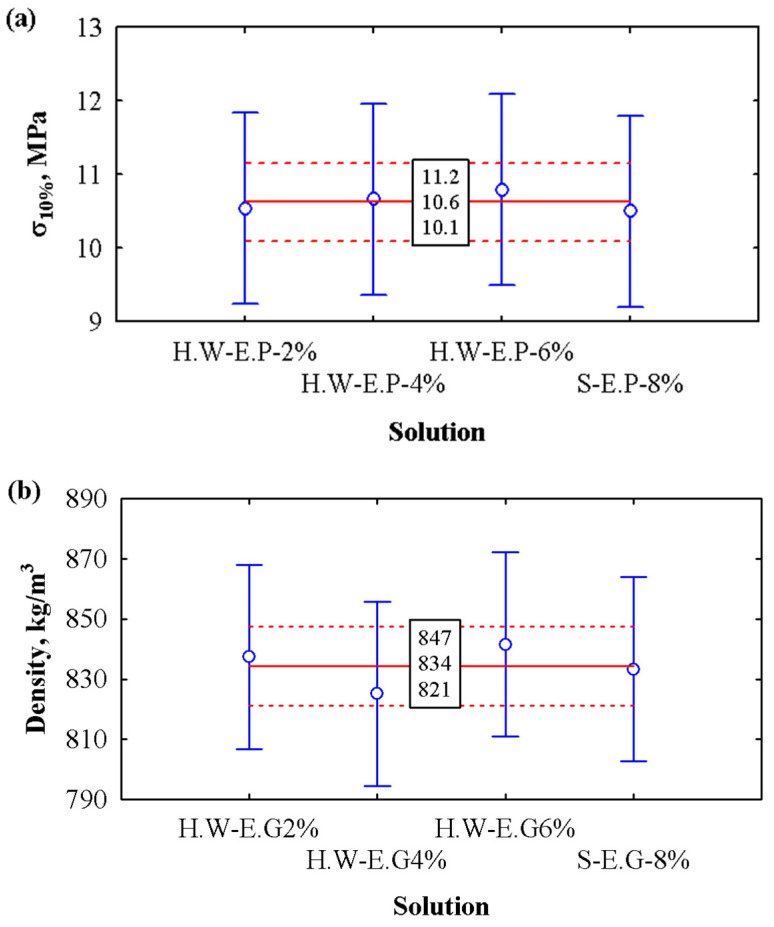
The influence of additives on the compressive strength and density of engineered wood composites: (**a**)—dependence on compressive strength; (**b**)—dependence on density; H.W-E.G-2%—hot water and 2% expandable graphite; H.W-E.G-4%—hot water and 4% expandable graphite; H.W-E.G-6%—hot water and 2% expandable graphite; S-E.G-8%—sodium carbonate and 2% expandable graphite (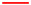
—mean value, 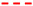
—confidence intervals, ○—average of composition results).

The analysis of the compressive stress of the composites (Figure 16a) showed that it does not depend on the treatment of straw with hot water and sodium carbonate or on the introduction of expandable graphite. Statistical analysis showed F = 0.054, *p* = 0.98, R = 0.142, and R^2^ = 0.0202.

The analysis of the compression tests showed that density also does not depend on the treatment of straw with hot water and sodium carbonate, or on the introduction of expandable graphite (Figure 16b). Statistical analysis showed F = 0.278, *p* = 0.84, R = 0.307, and R^2^ = 0.0944.

Since the *p*-value was >0.05, there was no statistically significant difference between the averages of the compressive stresses and densities for at least one group of composites made from straw particles treated with different solutions. This means that the type of solution and expandable graphite did not affect the compressive stress and density.

The following values were obtained after performing compression tests: hot water and 2% expandable graphite (10.5 ± 1.236 MPa—837 ± 28.7 kg/m^3^), hot water and 4% expandable graphite (10.7 ± 1.210 MPa—825 ± 29.8 kg/m^3^), hot water and 6% expandable graphite (10.8 ± 0.778 MPa—842 ± 14.3 kg/m^3^), and sodium carbonate and 8% expandable graphite (10.5 ± 0.474 MPa—833 ± 14.2 kg/m^3^).

Since the analysis showed no statistically significant difference between the test values, the compressive stress can be accepted as 10.6 ± 0.842 MPa and the density as 834 ± 20.6 kg/m^3^.

Statistical analysis of moisture properties was performed, showing a *p*-value < 0.05. This is a statistically significant difference in the averages of swelling, absorption, and density for at least one group of composites made from straw particles treated with different solutions. This means that the type of solution affected both swelling and water absorption, as well as density. The statistical data obtained from the swelling tests showed F = 50.4, *p* = 0, R = 0.975, and R^2^ = 0.950. The statistical data from the absorption tests showed F = 246.0, *p* = 0, R = 0.995, and R^2^ = 0.989, and those from the density tests showed F = 111.5, *p* = 0, R = 0.988, and R^2^ = 0.977.

**Figure 17:**
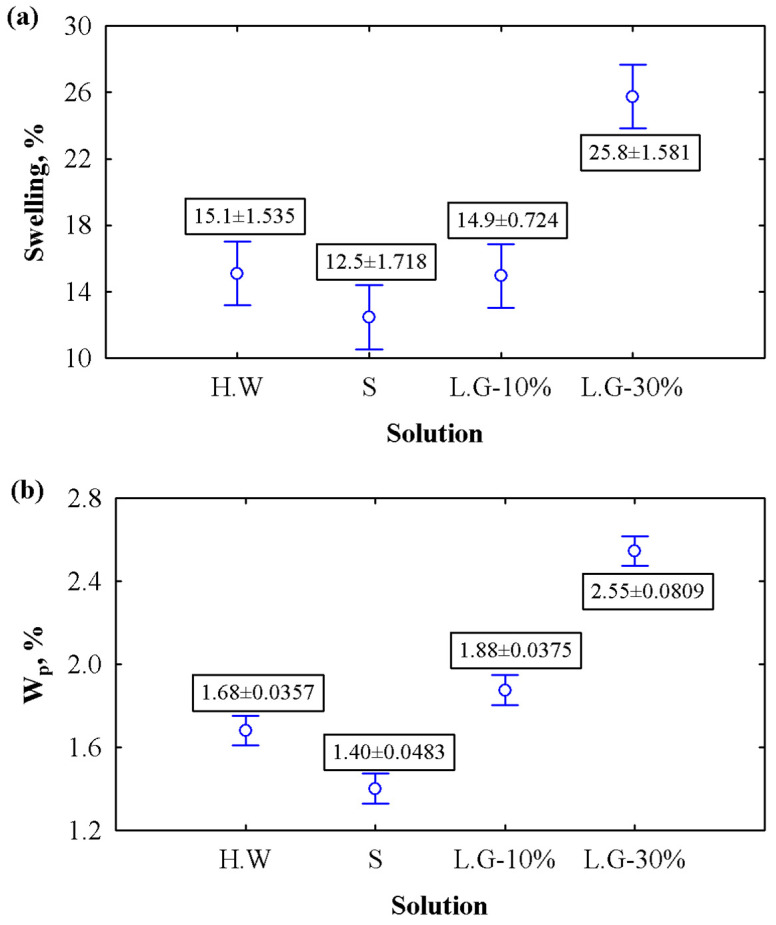

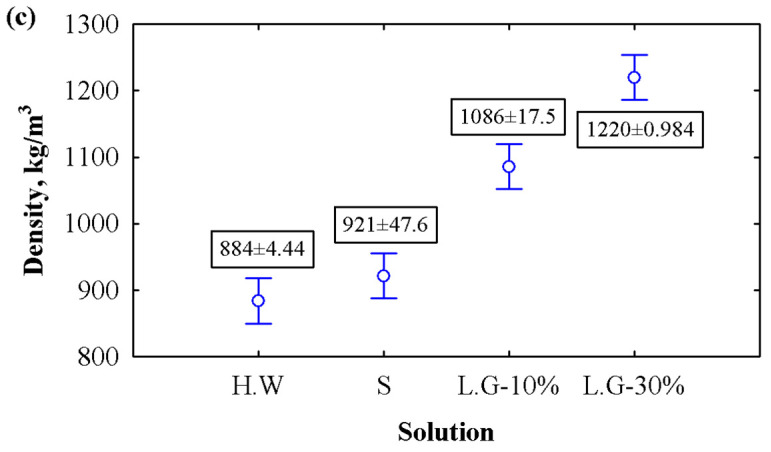
Studies of the moisture properties of engineered wood composites: (**a**)—swelling; (**b**)—water absorption; (**c**)—density; H.W—hot water, S—sodium carbonate, L.G-10%—10% liquid glass, L.G-30%—30% liquid glass.

Analysis of moisture properties showed that the lowest swelling and absorption were observed in the sodium carbonate-treated samples (Figure 17). Meanwhile, the greatest swelling and water absorption were observed in the composites whose surfaces were treated with the 30% liquid glass solution. It was found that the swelling difference between the sodium carbonate-treated and the 30% liquid glass was ~2.1 times. The difference in water absorption between the sodium carbonate-treated and 30% liquid glass was ~1.8 times, and the increase in density was ~1.3 times.

**Figure 18:**
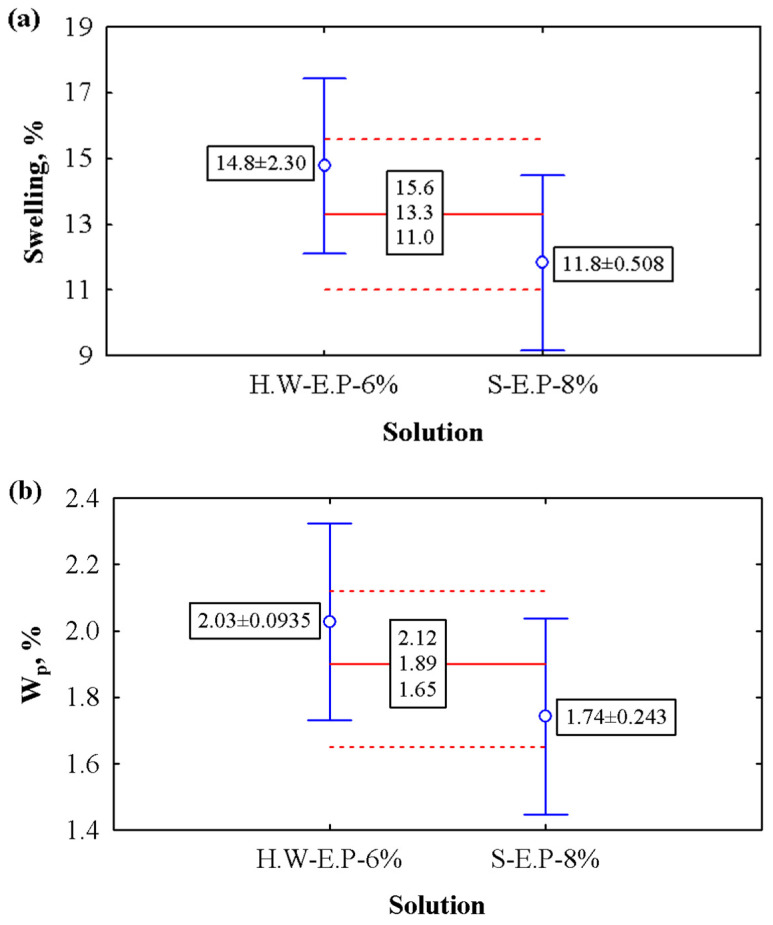

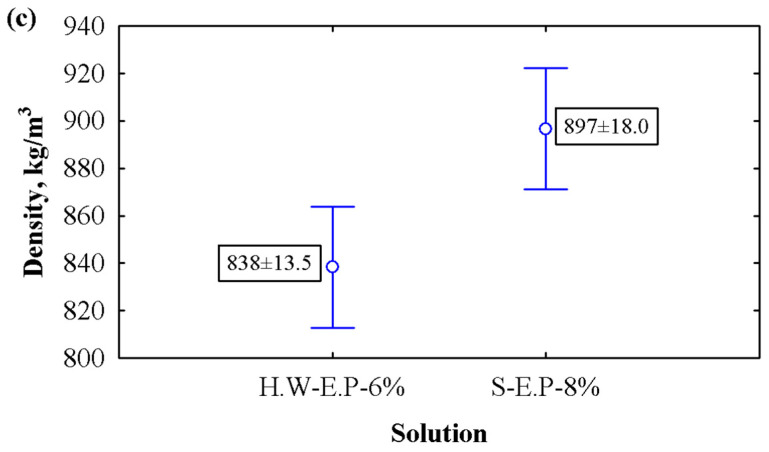
Studies of the moisture properties of engineered wood composites: (**a**)—swelling; (**b**)—water absorption; (**c**)—density; H.W-E.P-6%—hot water and 6% expandable graphite, S—sodium carbonate and 8% expandable graphite. (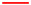
—mean value, 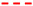
—confidence intervals, ○—average of composition results).

Statistical analysis of moisture properties showed that the *p*-values for swelling and absorption were >0.05. This indicates no statistically significant difference in the swelling and water absorption averages for at least one group of composites made from straw particles treated with different solutions. This means that the type of solution and expandable graphite did not affect either swelling or water absorption. The obtained swelling test statistics were F = 4.70, *p* = 0.096, R = 0.735, and R^2^ = 0.540, and for water absorption, they were F = 3.57, *p* = 0.13, R = 0.687, and R^2^ = 0.471. The analysis showed that the swelling and water absorption values did not differ; swelling was assumed to be 13.3 ± 2.20%, and water absorption was 1.89 ± 0.227%. Meanwhile, statistical analysis showed that the density *p*-value was < 0.05. This is a statistically significant difference between the average density of at least one group of composites made from straw particles treated with different solutions. This means that the type of solution and expandable graphite affected the density. The obtained density test statistics were F = 20.1, *p* = 0.011, R = 0.913, and R^2^ = 0.834. The analysis showed that the density values differ by ~7.0%. A graphical interpretation of test results for swelling and water absorption is presented in Figure 18.

**Figure 19:**
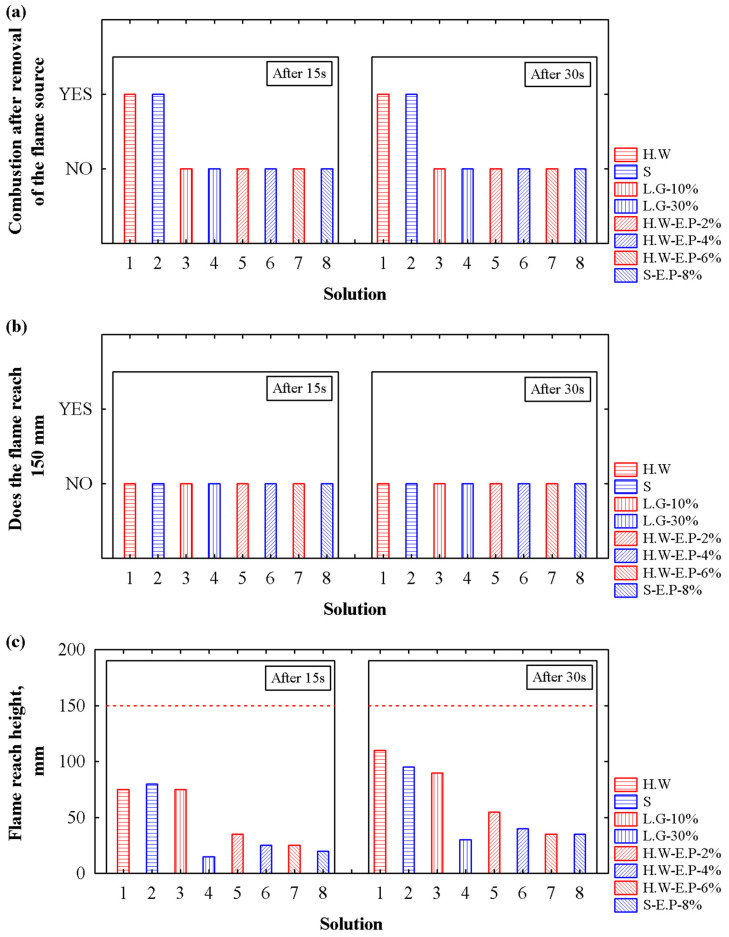
Flammability tests of a composite made of 5 mm straw particles treated with hot water and expandable graphite and sodium carbonate and expandable graphite: (**a**)—combustion after removing the flame source; (**b**)—whether the flame reaches 150 mm; (**c**)—flame height, mm.

Figure 19 shows the flammability results of an engineered wood composite treated with different solutions and with an expandable graphite additive. The test results showed that treating the straw particles with hot water and a sodium carbonate solution caused the composite to burn after the fire source was removed (Figure 19a). When evaluating the spread of fire on the surface of the sample, we determined that in none of the samples did the spread exceed the permissible limit of 150 mm after 15 or 30 s (Figure 19b). The lowest spread of fire was observed in the samples in which expandable graphite was used and in the samples in which the straw was treated with the 30% liquid glass solution (Figure 19c). When comparing the flammability of the samples formed with different amounts of expandable graphite and treated with the 30% liquid glass solution, we observed a fire spread of 20–50 mm, with the highest flammability in the samples containing 2% expandable graphite. This showed that 2% expandable graphite was insufficient to protect the sample surface from the effects of fire.

**Figure 20:**
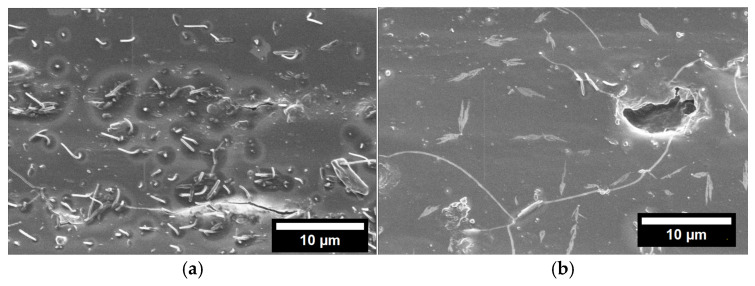

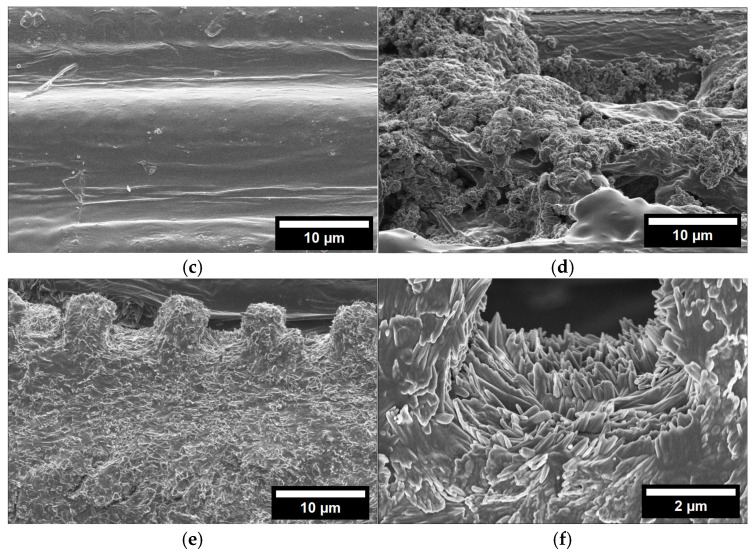
Surface view of straw particles: (**a**) untreated, ×5000; (**b**) treated with hot water, ×5000; (**c**) treated with sodium carbonate solution, ×5000; (**d**) treated with 10% liquid glass solution, ×5000; (**e**) treated with 30% liquid glass solution, ×5000; (**f**) treated with 30% liquid glass solution, ×25,000.

Figure 20 shows the surface of straw particles when straw was untreated and treated with different methods. The surface of untreated straw (Figure 20a) showed a natural biological microstructure dominated by a protective barrier with characteristic micro-formation elements. The entire surface of the straw particle was abundantly covered with micro-hairs and specific waxy coatings that protect it from various biotic and abiotic environmental factors [53]. The waxes, silica, lignin and hemicellulose layers on the straw surface hinder effective chemical adhesion and mechanical bonding with biopolyurethane [54,55]. The untreated straw surface has poor wettability, as indicated by higher water contact angles, which limits the spreading and penetration of adhesives [56]. The aforementioned micro-hairs and wax were partially removed by hot water treatment (Figure 20b). Hot water treatment alters the microstructure of straw by expanding its cells, which enhances mechanical interlocking with binding agents. This structural change improves the interfacial bond strength between the straw and the matrix material [57,58]. After treatment with soda carbonate, no signs of micro-hairs remained, and micro-irregularities appeared on the surface of the straw particle itself (Figure 20c). Typically, sodium carbonate treatment of straw removes or reduces these inhibitory surface components, increases surface roughness and porosity, and exposes more reactive functional groups (e.g., hydroxyl groups) that promote stronger adhesion to biopolyurethane. This dual effect—removing physical/chemical barriers and creating a more reactive, rougher surface—explains the improved adhesion of NaOH-treated straw to the biopolyurethane binder compared to untreated straw [55,59]. A double effect was observed when treated with a liquid glass solution. Depending on the concentration of the solution, active leaching and dissolution of various substances occurred on the straw surface and film formation on the straw surface (Figure 20d,e). Figure 20f shows that cellulose fibres were clearly visible on the surface of the straw particle after lignin was removed [60]. Although the adhesion of liquid glass to the surface of straw is not directly discussed in scientific articles, it is likely that its chemical properties, such as high surface energy, ability to form covalent bonds, and modification potential, contribute to better adhesion, and research in this area should be expanded in the future.

**Figure 21:**
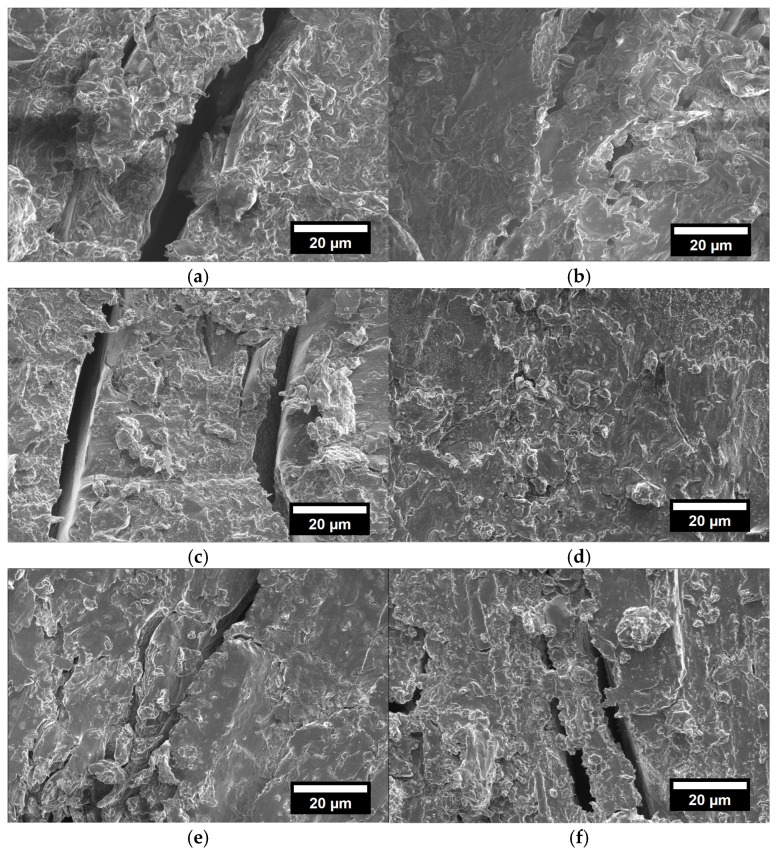
Surfaces of engineered wood composites made from differently treated straw at different compression levels and with varying amounts of binder; magnification ×2000: (**a**) untreated straw, binder ratio 0.5 and pressure level 1.5 MPa; (**b**) untreated straw, binder ratio 1 and pressure level 3.0 MPa; (**c**) hot-water-treated straw, pressure level 2.25 MPa and binder ratio 1; (**d**) sodium carbonate-treated straw, pressure level 2.25 MPa and binder ratio 1; (**e**) 10% concentration liquid-glass-treated straw, pressure level 2.25 MPa and binder ratio 1; (**f**) 30% concentration liquid-glass-treated straw, pressure level 2.25 MPa and binder ratio 1.

Figure 21 shows the surface of a composite made from differently treated straw at different compression levels and with varying binder content. Figure 21a,b present a general view of the samples formed with different amounts of binder. When untreated straw, a binder ratio of 0.5 (Figure 21a) and a compression level of 1.5 MPa were used for sample preparation, large voids were observed in the sample structure. This shows that the binder was insufficient at completely filling all the gaps between the straw particles and that the compression level was too low because the particles were compressed too little relative to each other. When the compression level was increased to 3 MPa and the polyurethane binder ratio was 1.5 (Figure 21b), large voids no longer formed in the structure of the engineered wood composite, but only individual air cells. When hot-water-treated straw was used for sample preparation, the compression level was 2.25 MPa, and the binder ratio was 1 (Figure 21c). The gaps between the straw particles were reduced, but weak contact zones left smaller voids. When soda carbonate-treated straw was used for sample preparation, the binder ratio was 1 (Figure 21d), and the compression level was 2.25 MPa; neither large voids nor single air cells were observed in the sample structure. The corresponding images of the samples prepared from straw treated with liquid glass at 10% and 30% concentrations are shown in Figure 21e,f. Single voids were again observed on the surface of the samples. It is likely that these voids did not result from poor contact zones between the straw particles and the polyurethane binder, but rather from technological factors. Straw particles treated with liquid glass become significantly harder, making them difficult to compress during sample preparation, thereby leaving individual voids.

Engineered wood products are commonly used in construction and furniture for various purposes—plywood [61], oriented strand boards [62], particleboards [63,64], fibreboards [65], and wood–plastic composites [66]. Thus, their application areas and test methods are described in European standards. According to the requirements of the standards [63,64,66], these products are made from shredded wood or other plant parts—hemp, flax, sisal, coconut, cotton, kenaf, jute, abaca, banana leaf fibres, bamboo, rice, wheat straw or other fibrous materials and binding materials. Thus, the name “engineered wood” does not fully indicate the product’s origin; it only identifies its product group. In addition, when reusing recycled wood, the amount of wood, the number of other plants, and their ratio in the mixture are usually not assessed. There are more ambiguities in this product group. The above standards only cover thin products; in all cases, thermal treatment is used to harden the binder, and thicker products are produced only by additional glueing. This production method determines that only the bending strength test is relevant for thin products. Our engineered wood made from straw and biopolyurethane differs from other engineered wood composites in that it does not require thermal treatment and can be made with a practically unlimited thickness. This indicates that our composite can be used to produce blocks, large-sized panels, or engineered wood logs, and that the most relevant property in this case is compressive strength rather than bending strength. Since the products are made with a plant-based filler, it is also relevant to assess their resistance to fire and water.

In summary, the performance of the polyurethane binder/straw engineered wood composite was determined by the interface between the straw particles and the polyurethane binder matrix. Mechanical and chemical treatment of the straw particles, selection of the binder amount, and the level of mixture compression increased the compressive strength of the composite from 3.84 MPa to 17.0 MPa. For comparison, the compressive strength of natural coniferous wood across fibres ranges from 4.3 to 6.3 MPa [67], and the compressive strength of building blocks with wood filler, when the wood–cement ratio ranges from 0.905 to 1.54, ranges from 0.64 to 12.27 MPa [68]. In other scientific studies [69], the amount of polyurethane binder in the engineered wood was set to range from 40 to 60%, and the aggregate was sieved through 2 mm sieves. Increasing the binder content increases bending strength by up to 1.2 times and tensile strength by about 3 times. Other authors evaluated the influence of filler particle aspect ratio on compressive strength [70]. The authors found that when the aspect ratio is 3, the compressive strength is highest, but they did not evaluate the filler particle size fraction, filler particle density, or composite density. Ding et al. [71] studied the influence of hot alkali-treated wheat, corn, and rice straw on the strength, density, and water absorption of a composite. The authors found that chemical treatment improves the composite’s properties, but the choice of straw type has a significant impact. The choice of binder, as noted by Boquillon et al. [72], has a significant impact when straw is used. The surface free energy of straw has a much lower polar component than that of wood species, so they are more compatible with oil resins than with water-soluble systems. For this reason, the choice of polyurethane binder for the preparation of engineered wood composites with barley straw is justified.

## 4. Conclusions

1. Straw processing methods determine the properties of both the straw itself and the engineered wood composite. The most suitable fraction of the studied straw for producing engineered wood composites is 5 mm. This work did not additionally investigate the possibility of using different varieties of barley or other types of straw, but this could be relevant to research in the future, since the use of straw for the production of engineered wood composites has reasonable potential.
2. Proper selection of the amount of polyurethane binder and the level of compression of the composite mixture allows for the formation of a homogeneous product structure and regulation of the operational properties of the composite.
3. The flammability, absorption and swelling of engineered wood composites are greatly influenced not only by the amounts of additives used but also by the formation of the composite structure, which is related to the straw processing methods, the choice of the amount of binder and the level of compression of the mixture.

The results obtained in this work show that barley straw can be used to produce engineered wood products with a polyurethane binder. In the future, it is necessary to improve methods for processing straw itself and to test additional additives that enhance properties and reduce the amount of polyurethane binder, the most expensive component of the composite from an economic standpoint.

## Figures and Tables

**Table 1:** Results of straw particle shape analysis.

Fraction, mm	Residue on the Sieve, mm	Aspect Ratio	Roundness	Circularity
5	0	4.207	0.316	0.448
	2.5	6.401	0.199	0.31
	5	6.376	0.181	0.089
10	0	3.544	0.378	0.432
	2.5	4.535	0.274	0.216
	5	5.945	0.2	0.172
	10	5.944	0.198	0.202
25	0	2.544	0.439	0.533
	2.5	5.578	0.21	0.337
	5	5.489	0.23	0.231
	10	6.231	0.23	0.183
	20	11.707	0.124	0.159
35	0	3.718	0.342	0.366
	2.5	3.897	0.272	0.308
	5	6.899	0.179	0.206
	10	8.317	0.175	0.124
	20	8.383	0.157	0.127
	25	12.889	0.12	0.078

**Table 2:** Statistical results of the data used to determine the density of straw particles of different fractions.

Equation	Number of Samples	R	R^2^	S_r_
ρ→pressure	$\rho =119.9856+0.01352\cdot pressure+\frac{-711.007}{prressure}$ (5) *			
	18	0.989	0.978	2.150
	$\rho =59.36895+0.01005\cdot pressure+\frac{-443.992}{pressure}$ (6) **			
	18	0.986	0.972	1.710
	$\rho =72.4893+0.01073\cdot pressure+\frac{-551.621}{pressure}$ (7) ***			
	18	0.994	0.988	1.210
	$\rho =52.77493+0.009622\cdot pressure+\frac{-333.611}{pressure}$ (8) ****			
	18	0.863	0.745	5.290

Note: * 5 mm; ** 10 mm; *** 25 mm; **** 35 mm fractions.

**Table 3:** Statistical results for the mechanical properties of a composite made from a 5 mm straw fraction.

Equation	Number of Samples	R	R^2^	S_r_
ρ→ amount of binder	$\rho =259.4190+692.3418\cdot amount\;of\;binder-200.483\cdot {amount\;of\;binder}^{2}\;$ (10) *			
	15	0.928	0.861	40.0
	$\rho =21.40661+1312.172\cdot amount\;of\;binder-499.710\cdot {amount\;of\;binder}^{2}\;$ (11) **			
	15	0.881	0.776	31.7
	$\rho =88.42593+1321.935\cdot amount\;of\;binder-537.108\cdot {amount\;of\;binder}^{2}\;$ (12) ***			
	15	0.957	0.916	35.3
$\sigma_{10\%}\to \rho$	$\sigma_{10\%}=-13.3028+0.02715\cdot \rho$ (13) *			
	15	0.993	0.986	0.387
	$\sigma_{10\%}=-13.1108+0.02777\cdot \rho$ (14) **			
	15	0.969	0.938	0.961
	$\sigma_{10\%}=-12.8141+0.02640\cdot \rho$ (15) ***			
	15	0.984	0.969	0.555

Note: * 1.5 MPa; ** 2.25 MPa; *** 3.0 MPa pressure.

**Table 4:** Statistical results of the data used to determine the swelling and water absorption properties of a composite made from 5mm fraction straw particles.

Equation	Number of Samples	R	R^2^	S_r_
ρ→ amount of binder	$\rho \;=\;\;79.31959+1049.958\cdot amount\;of\;binder-360.803\cdot {amount\;of\;binder}^{2}\;$(16) *			
	15	0.982	0.965	26.0
	$\rho \;=\;\;179.9690+992.6888\cdot amount\;of\;binder-348.944\cdot {amount\;of\;binder}^{2}\;$(17) **			
	15	0.976	0.953	27.6
	$\rho \;=\;\;191.0859+1167.341\cdot amount\;of\;binder-479.620\cdot {amount\;of\;binder}^{2}\;$ (18) ***			
	15	0.988	0.977	15.4
swelling→ρ	IŠB=76.97056−0.0853·ρ (19) *			
	15	0.977	0.954	2.51
	IŠB=75.15077−0.0774·ρ (20) **			
	15	0.965	0.931	2.56
	IŠB=114.5192−0.1164·ρ (21) ***			
	15	0.954	0.911	3.53
$W_{p}\to \rho$	$W_{p}=15.32581-0.0171\cdot \rho$ (22) *			
	15	0.992	0.984	0.293
	$W_{p}=15.5190-0.0165\cdot \rho$ (23) **			
	15	0.977	0.955	0.438
	$W_{p}=17.79268-0.0184\cdot \rho$ (24) ***			
	15	0.981	0.963	0.349

Note: * 1.5 MPa; ** 2.25 MPa; *** 3.0 MPa pressure.

**Table 5:** Statistical results of the density dependence of 5mm fraction straw particles after treatment with various solutions on loading properties.

Equation	Number of Samples	R	R^2^	S_r_
ρ→pressure	$\rho =92.81609+0.006124\cdot pressure+\frac{-516.954}{pressure}$ (25) *			
	18	0.936	0.877	2.894
	$\rho =85.70949+0.006378\cdot pressure+\frac{-534.312}{pressure}$ (26) **			
	18	0.975	0.950	1.828
	$\rho =176.6576+0.006443\cdot pressure+\frac{-709.431}{pressure}$ (27) ***			
	18	0.943	0.888	3.278
	$\rho =240.3003+0.009712\cdot pressure+\frac{-936.052}{pressure}$ (28) ****			
	18	0.985	0.971	2.247

Note: * sodium carbonate, ** hot water, *** 10% liquid glass, and **** 30% liquid glass.

## Data Availability

The original contributions presented in this study are included in this article. Further inquiries can be directed to the corresponding author.
